# Barriers to the uptake of community-based curative child health services in Ethiopia

**DOI:** 10.1186/s12889-021-11558-2

**Published:** 2021-08-14

**Authors:** Birkety Mengistu, Meron Paulos, Nesibu Agonafir, Agazi Ameha, Hailemariam Legesse, Elizabeth Dankenbring, Mariame Sylla, Nathan P. Miller

**Affiliations:** 1 PATH, Addis Ababa, Ethiopia; 2 UNICEF, Addis Ababa, Ethiopia; 3 UNICEF, Freetown, Sierra Leone; 4 Columbia University Mailman School of Public Health, New York, USA; 5 UNICEF, Pretoria, South Africa; 6 UNICEF, New York, USA

**Keywords:** Barrier analysis, Community-based newborn care, Health extension program, Health extension workers, Integrated community case management, Ethiopia

## Abstract

**Background:**

Uptake of services to treat newborns and children has been persistently low in Ethiopia, despite being provided free-of-charge by Health Extension Workers (HEWs). In order to increase the uptake of these services, the Optimizing the Health Extension Project was designed to be implemented in four regions in Ethiopia. This study was carried out to identify barriers to the uptake of these services and potential solutions to inform the project.

**Methods:**

Qualitative data were collected in October and November 2015 in 15 purposely selected districts in four regions. We conducted 90 focus group discussions and 60 in-depth interviews reaching a total of 664 participants. Thematic analysis was used to identify key barriers and potential solutions.

**Results:**

Five demand-side barriers to utilization of health services were identified. Misconceptions about illness causation, compounded with preference for traditional healers has affected service uptake. Limited awareness of the availability of free curative services for children at health posts; along with the prevailing perception that HEWs were providing preventive services only had constrained uptake. Geographic challenge that made access to the health post difficult was the other barrier.

Four supply-side barriers were identified. Health post closure and drug stock-out led to inconsistent availability of services. Limited confidence and skill among HEWs and under-resourced physical facilities affected the service delivery.

Study participants suggested demand creation solutions such as increasing community awareness on curative service availability and educating them on childhood illness causation. Maintaining consistent supplies and ensuring service availability; along with regular support to build HEWs’ confidence were the suggested supply-side solutions. Creating community feedback mechanisms was suggested as a way of addressing community concerns on the health services.

**Conclusion:**

This study explored nine demand- and supply-side barriers that decreased the uptake of community-based services. It indicated the importance of increasing awareness of new services and addressing prevailing barriers that deprioritize health services. At the same time, supply-side barriers would have to be tackled by strengthening the health system to uphold newly introduced services and harness sustainable impact.

**Supplementary Information:**

The online version contains supplementary material available at 10.1186/s12889-021-11558-2.

## Background

Ethiopia has achieved Millennium Development Goal 4 (MDG 4), decreasing child mortality by more than two-thirds [1]. However, 188,690 children under-5 died in 2018 and neonatal deaths accounted for more than 40% of these deaths [2]. Many of these deaths could have been averted through high-impact interventions at the community level which includes appropriate diagnosis and treatment with antibiotics, anti-malaria, oral rehydration salt and zinc [3].

**Fig. 1:**
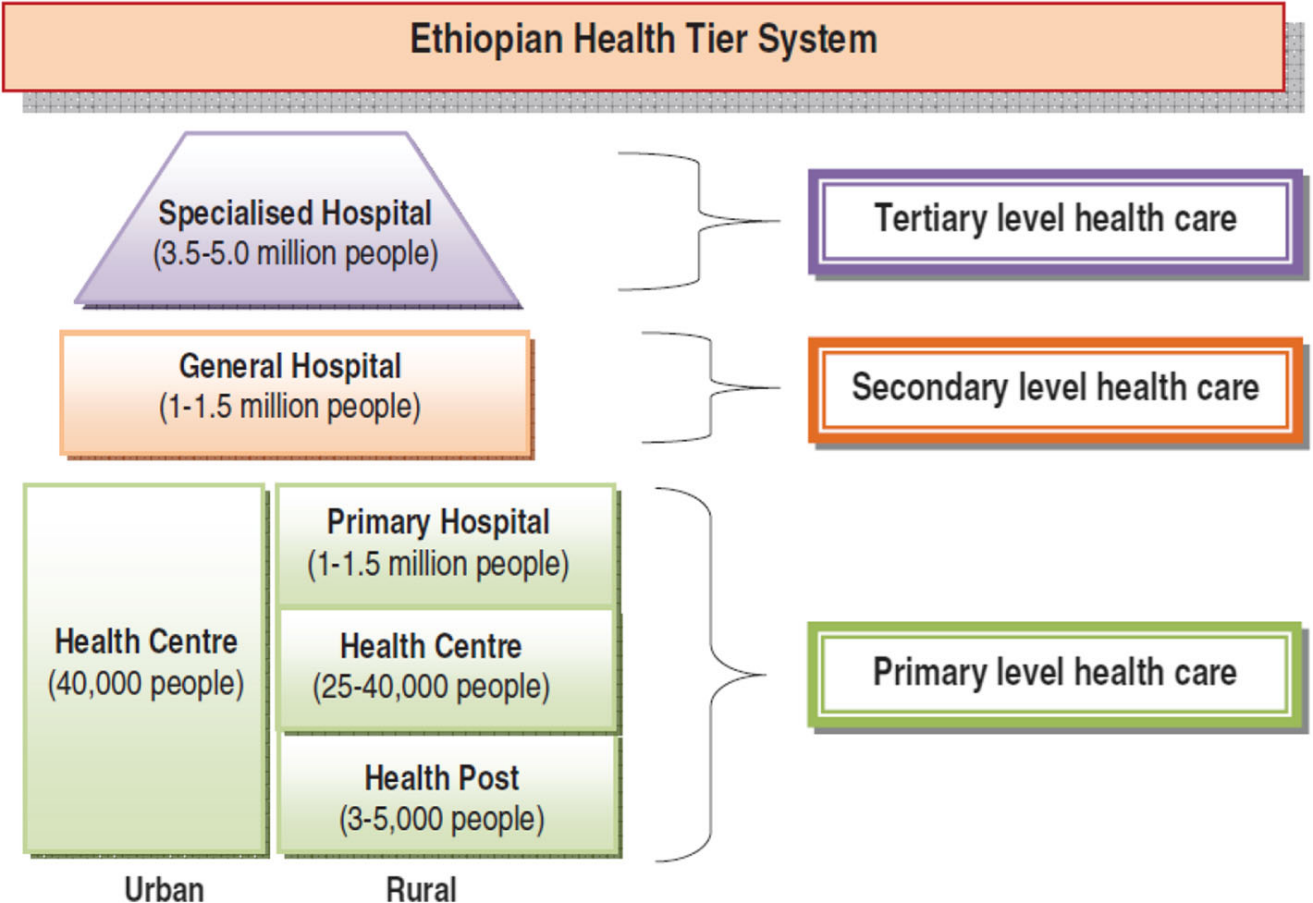
The healthcare system of Ethiopia

The Ethiopian primary healthcare system has a three-tiered structure consisting of primary hospitals, health centers and health posts as the first-tier, general hospitals make the second tier and specialized hospitals are at the tertiary tier (Fig. 1). The frontline facilities are health posts, which serve an average catchment population of 5000 people. Health posts are staffed with two female Health Extension Workers (HEWs). HEWs are high school graduates with one year of practical and theoretical training on health extension packages (HEP) and are salaried by the government [4]. The original HEP included 16 preventive, promotive and basic curative packages, which grew to 18 packages in subsequent years [5, 6]. Health posts provide services on weekdays between 8:00 a.m. and 5:00 p.m. and HEWs are expected to provide emergency services during off-duty hours, as they are expected to reside within the premise of the health posts. When the program started, HEWs were expected to spend 75% of their time providing outreach and house-to-house services [7]. This was later reduced to 50% to enable provision of services from the health post. When one of the HEWs goes out to the field, the other would be able to provide services from the health post.

In 2010, the Federal Ministry of Health (FMoH) and partners scaled-up integrated community case management (iCCM) in four regions of the country, namely Amhara, Oromia, Tigray and Southern Nations, Nationalities and Peoples (SNNP) Regions [8]. Under the iCCM program, sick children receive oral rehydration solution (ORS) and zinc for diarrhea, antibiotics for pneumonia, artemisinin-based combination therapy (ACT) or chloroquine for malaria, and ready-to-use therapeutic food and amoxicillin for severe acute malnutrition.

To achieve coverage of quality iCCM services, cascaded competency-based training was provided for HEWs, followed by supportive supervision and performance reviews and clinical mentorship. iCCM commodities were directly given as starter kits to HEWs following the initial iCCM training. The commodities continued to be directly provided by implementing partners to the health posts, until they were integrated into the national pharmaceutical logistics system [8].

A new cadre of community volunteers known as the Women’s Development Army (WDA) was introduced in 2011. Leaders of the WDA were trained by the HEWs on community health promotion and the creation of “model households” and reported their activities back to the HEWs [9]. The *kebele* (sub-district) administration, known as *kebele* cabinet, includes one of the HEWs and provides oversight to the WDA leaders. The WDA initiative is estimated to involve approximately 3 million women [10].

As a way of combating the newborn mortality rate, which did not show substantial reduction,^1^ community-based newborn care (CBNC) was introduced in 2013 using the iCCM platform. CBNC uses a “four Cs” approach along the continuum of maternal and newborn care: prenatal and postnatal **c**ontact with the mother and newborn; **c**ase identification of newborns with signs of possible severe bacterial infection; **c**are or treatment that is appropriate and initiated as early as possible; and **c**ompletion of a full seven-day course of amoxicillin and gentamicin for those who refused referral to the health center. The training, supervision and rollout followed a similar approach to iCCM [11].

An independent evaluation of the scale-up of iCCM in Oromia Region found that despite the relatively high program implementation strength and quality of care, low utilization of services limited its impact [12–14]. This concurs with findings from other studies. For example, a study that reviewed annual data of iCCM registers from 622 health posts found that HEWs treated only 8% of expected pneumonia cases and 1% of diarrheal cases [15]. A qualitative study in Oromia region on care-seeking behavior found a number of demand- and supply-side barriers to utilization of iCCM services, including lack of availability of HEWs at the health post, financial and geographic challenges, perceived lack of sensitivity of HEWs and concerns about medicines given at the health post [16].

Optimizing the Health Extension Project (OHEP) was a three-year project (July 2015 to June 2018) funded by the Bill & Melinda Gates Foundation to be implemented by PATH^2^ and UNICEF^3^ which aimed to increase iCCM and CBNC utilization. At the beginning of the program, in late 2015, a qualitative study on barriers to utilization of iCCM and CBNC services was conducted to inform the development of evidence-based and context-specific interventions. This study assessed the demand- and supply-side barriers and enablers at the family/household, community, and health system levels.

## Methods

### Study design

Exploratory qualitative research was conducted to identify barriers to community-based curative newborn and child health service utilization and to identify potential solutions to these barriers.

### Study areas

The study was conducted in Tigray, Amhara, Oromia and SNNP Regions,^4^ where 75% of the Ethiopian population resides and which are dominantly agrarian. The study was conducted in the Awi Zone of Amhara, Guji and the West Harerge Zones of Oromia, the South Eastern Zone of Tigray and the Gurage Zone of SNNP, which were selected as the project intervention zones by the regional health bureaus mainly for their relatively low performance within their regions. According to the 2007 census, the total population in the four zones was projected to be about 6 million in 2015.

The study was conducted in 15 districts (Table 1). A mix of high (four) and low (11) performing districts were purposively included in the study to increase representativeness. High- and low-performing districts were identified based on their sick newborn and child caseload from the routine service data obtained through the health management information system and a performance ranking made by the respective zonal health offices. Four high performing districts, one from each region, were purposely selected to identify best practices that may have led to increased uptake of services. A *kebele* was selected from each district taking the highest-performing *kebele* from the higher-performing district and the lowest-performing *kebele* from the lower-performing district.

**Table 1:** Study area

Region	Zone	Number and name of sampled districts
Amhara	Awi	4 (Banja, Fagta, Ankesha, Jawi^a^)
Oromia	Guji	2 (Wadera, Saba-Boru)
	West Hararge	2 (Boke, Doba^a^)
SNNP^b^	Gurage	4 (Geta^a^, Meskan, Gummer, Abeshige)
Tigray	South Eastern	3 (Enderta, Degua Tembien^a^, Hintalo Wajirat)
Total	5	15

^a^Higher-performing districts

^b^Southern Nations, Nationalities and Peoples Region

### Study population and sampling

Study participants were purposively selected bearing in mind their positions and knowledge of the subject area under research. Parents whose under five children were sick in the preceding month were included to understand care-seeking patterns. To encourage openness during the FGDs, separate sessions were conducted with mothers and fathers. To validate the FGDs, additional three in-depth interviews (IDIs) were conducted with mothers, where one was with a mother who sought care for her sick child. Influential community members (religious leaders, traditional healers, clan leaders) were reached through FGDs as they are mostly engaged in treating sick children or in providing advice for parents.

WDA leaders were included in the FGDs given their role in health promotion within the community. *Kebele* cabinet members were part of the FGDs as they oversee the service of HEWs and the WDA. HEWs were included in the study to understand their perspective on iCCM and CBNC services. In kebeles where there were two HEWs, one of the HEWs participated in an IDI, while the other participated in the FGD of the *kebele* cabinet members. District health stakeholders – including the district health office head, the health center directors, the district maternal and child health (MCH) coordinator, the district HEW coordinator, and developmental partners working in MCH in the district – were included at the district level. Six focus group discussions (FGDs) (with mothers, fathers, influential community members, WDAs and *kebele* cabinet members and district stakeholders) and four in-depth interviews (IDIs) with mothers and HEWs were performed per district (Table 2). A total of 90 FGDs and 60 IDIs were conducted, reaching 664 participants in 15 districts.

**Table 2:** Data collection methods and participants per districts

Participant Type		Number of In-depth Interview per district	Number of focus group discussion per district	Average number of participants	Total number of participants	Age range
Mothers	Who did not seek care for their sick children from health post	2	1	8.4	126	18–48
	Who sought care for their sick children from health post (exit interview whenever possible)	1		1	15	20–65
Fathers			1	8	120	20–62
*Kebele* (sub-district) Cabinet Members			1	6.3	95	22–55
Health Extension Workers		1		1	15	21–36
Women Development Army Leaders			1	7.8	117	18–69
Community leaders			1	6.6	99	25–90
District level stakeholders			1	5.1	77	22–54
Total		4	6	44.4	664	18–90

Community-level sampling: To ensure objective recruitment of parents, agriculture extension workers were used to identify participants instead of HEWs or WDA leaders. HEWs and WDA leaders could be biased in selecting those that are closer to the health service than the general population. The agriculture extension workers had a relatively neutral position and since they live within the community, they were able to support the recruitment of participants for the data collectors.

### Data collection

FGD and interview guides were developed specifically for each type of informant (Additional file 1) and translated into three local languages (Tigrigna, Afan Oromo and Amharic^5^). The discussion questions focused on the general health status of children, care seeking patterns, barriers to seeking care from the health post and potential solutions. Data collectors were experienced public health professionals with master’s degrees who spoke the respective local language. They received a two-day intensive training on the tools prior to collecting data. The instruments were pre-tested in the respective regions before data collection and appropriate corrections to the tools were made.

Data were collected in October and November 2015. A data collection team with two data collectors was deployed in each of the study zones. In each zone, the two data collectors conducted IDIs independently. During FGDs, one served as a facilitator and the other as a note-taker. Consultants from HANDS ON Research and Training PLC, a private Ethiopian research firm, and project staff members from PATH and UNICEF carried out field supervision. All interviews were audio-recorded and additional notes were taken for documenting expressions, interesting quotes and main points.

### Data management and analysis

Audio recordings were transcribed by the data collectors and the transcripts were then translated into English by the data collectors. Transcripts were then exported to NVivo software, where they were coded and categorized into predetermined and emerging themes. Two researchers conducted the data analysis by ensuring consistency of the transcribed data with the audio recordings. They first categorized the data using deductive thematic analysis by using predetermined codes [16]. The predetermined codes were demand- and supply-side barriers and potential solutions. We used both the inductive and the deductive approach in our study. We had deductively set to identify the demand and supply side barriers and potential solutions as these were what we were looking for to answer from our study. Within these large and overarching categories, we then inductively determined the lower-level themes. When an emerging theme was identified, the two analysts discussed it thoroughly before reaching agreement on how to code and categorize it inductively [17].

## Results

### Demand-side barriers

Five demand-side barriers that contributed to low uptake of iCCM and CBNC services were identified. These include myths and misconceptions around the cause of childhood illnesses including low risk perception, preference for traditional healers or home remedies, lack of awareness of iCCM and CBNC services, perceived poor quality of care and lack of transport and distance to health post.

#### Myths and misconceptions around the causes of childhood illness

Although recognition of the symptoms of childhood illnesses was found to be high, there were important misconceptions on the causes which had implications on care-seeking practices. This included attributing illnesses to ‘*mich*’ (exposure to sunlight while breastfeeding or eating or exposure to a dirty environment or bad smell), cold weather and ‘evil eye’. An FGD participant mother who did not seek healthcare from Tigray explained:

*“Some diseases, like diarrhea, cough and fever are caused as a result of exposing our children to direct sunlight.”*

In Oromia, newborn illnesses were thought to be caused by the shadow of a bird flying over the mother when she was pregnant: “*A bird (*‘*allati*’*) flying over a pregnant mother will make her malnourished, and her newborn baby will be weak,*” a community leader explained in FGD.

Misconceptions were also shared by the WDA leaders.

*“If children are breastfed from a pregnant mother, the clots from the milk will cause generalized body swelling.”* (WDA leader, Oromia)

If a malevolent spirit or evil eye were believed to be the cause of an illness, biomedical treatment was not seen as appropriate.

*“If a person assumes the child got the illness due to ancestral spirits, he will not bring the child to the health post. That child should be treated at home. This is called ‘ye bet tenk’ (ancestral problem).”* (Community leader, Amhara)

Similarly, some illnesses such as ‘yewof beshita’ (disease characterized by yellowish discoloration of the skin) and measles were widely believed to have unknown causes that could not be treated with allopathic medicine. In fact, these illnesses were considered to get worse with biomedical treatment.

##### Low risk perception

Most participants indicated that perceived severity of an illness determined the level of urgency with which they sought care for their children. Few mothers sought care for their sick children immediately after the onset of the illness, while most sought care only after the disease persisted for two to three days. This was attributed to the belief that childhood illnesses were generally self-limiting.

#### Preference for traditional healers or home remedies

Closely related with the misconception of causes of illness, the preference for traditional healers and use of home remedies were demand-side barriers in all four regions. Most participants in this study indicated that they use home remedies and/or traditional medicine as their first choice of care, prior to seeking care at the health post.

Failure to breastfeed among newborns was thought to be caused by a problem in the “tonsils” (uvula), for which the uvula was removed by traditional healers.

*“When a newborn is unable to suck breast milk or has vomiting and fever, we will take the newborn to a well-known traditional practitioner. Once the tonsil is cut, the child will regain his health and will start sucking.” (*Mother who did not seek care, FGD participant, Oromia)

Caregivers reported using herbs and plant leaves to treat their children. Delays in seeking care were reported as a result of caregivers’ habit of trying different treatments from different sources of care.

*“When children are sick, parents try traditional treatment first, such as taking them to a ‘tenkway’ [witch] … or use of ‘damakese’ [Chenopodium-herbal treatment for ‘mich’] ...These are the most common practices in our community.”* (*Kebele* cabinet member, Amhara)

Commonly reported home remedies included the juice of a herb known locally as ‘*damakese*’ for ‘*mich*’; garlic and ginger boiled together for presumed throat problems; a mix of salt with water along with burning the abdomen with hot metal for diarrhea; and lemon, salt and ‘*tenadam*’ (Rue/Ruta graveolens) for malaria. Self-medication with a single dose of antibiotics was also reported.

*“My child had a cough and was vomiting. I did not take her to a health facility. I have never taken her to a health facility. I always use traditional medicine. I gave her boiled garlic and ginger with sugar, and she got better." (*Mother who did not seek care, FGD participant, SNNP)

Participants in all the four regions indicated that slaughtering animals with certain rituals was used as a healing practice when a newborn or a child was sick.

*“If a newborn cry continuously, they move a red colored chicken around the newborn’s head and throw it...or they give him traditional medicine for evil eye or religious leaders pray for the newborn continuously for 15 days.”* (Mother who did not seek care, FGD participant, SNNP)

Participants from Awi, Amhara indicated that the communicable nature of ‘*ema wetete’* (local term for measles) necessitated traditional ritual. A mother described this ritual healing process during an FGD:

*“We make coffee, boil some cereals, and bake bread and ‘injera’ [the local staple food]. Once ‘ema wetete’ tastes the food and coffee, she will leave our neighborhood … We do not need to take the child to a health facility.”* (Mother who did not seek care, FGD participant, Amhara)

Additionally, some personal and spiritual beliefs indicated that the consumption of medicine was contradictory to their belief systems. Moreover, some illnesses were assumed to have ‘permanent cure’ only with traditional medicine or rituals.

*“It is different from any other diarrhea, where the child had offensive diarrhea and he cried when peeing...When we take such children to health facilities, they may get relief for a few days and then they would get sick again. But if they are treated with traditional treatment called ‘arek’ [dried root of a plant locally known as ‘arek’]* t*hey will never get sick again with the same problem.”* (Father who did not seek care, SNNP)

#### Lack of awareness of iCCM and CBNC services

Lack of awareness of the availability of curative child health services at the health post was a top demand-side barrier in all regions. Most caregivers considered HEWs as providers of preventive services only. Those who knew of the availability of curative services did not know that the services were free of charge. In particular, availability of treatment for sick young infants and for pneumonia were not well known by the participants. A mother whose child was sick, but did not seek care from the health post, indicated the following:

*“I do not know the services given at this health post … I have taken children [to the health post] for vaccination and for nutritional supplements only.”* (IDI with mother who did not seek care, Oromia)

The functionality of the WDA structure which was expected to increase awareness of iCCM and CBNC services in the community was mostly questionable. Most of the respondent mothers were unable to identify the WDA leader in their community and some of the WDA leaders revealed that they did not have regular meetings with the HEWs. Furthermore, many WDA leaders were not aware of the spectrum of HEWs’ services.

*“There is no treatment for cough [at the health post]. The HEWs refer mothers who come to this health post to the health center immediately.”* (WDA leader, Amhara)

Respondents also noted concerns about costs in seeking care as they were not aware that these services were provided for free at the health post.

*“Yes, my children are sick even today...I cannot take them to the health center. I have no money.”* (FGD participant mother who did not seek care, Oromia)

#### Perceived poor quality of care

Even though most community participants attributed the children’s improved health to immunization services and health education provided by HEWs, their curative skills were not well recognized. The perception that HEWs delivered poor-quality care emerged either from an assumption that HEWs were not qualified enough to provide curative services or from past negative experiences. Mothers who took their children to a health post and did not get appropriate care or faced stockouts reported that they decided not to go back to the health post again. In addition, mothers who were referred by HEWs to a health center without clear explanation, or who were mistreated, reported that they did not want to go back to that health post:

*“HEWs do not know how to treat childhood illnesses. For example, I was told by health educators to take my children to the health post if they get sick. But when I took my baby because of fever and vomiting, they said they have no medication. I think it is not only lack of medication; they may not know how to treat babies*.*”* (Mother who did not seek care, FGD participant, Oromia)

Problems related to physical structures were also raised as important deterrents to care seeking. For example, lack of furniture, poor physical structures and the untidiness of the health post contributed to the perceived poor quality of care at the health post by some caregivers.

#### Lack of transport and distance to health posts

Distance to health posts and lack of transportation were found to affect caregivers’ ability to access health posts. In almost all the districts, participants reported that the catchment area was large, and villages were dispersed, making access to the health posts difficult for people living on the outskirts of the kebeles. Difficult topography and terrain were also reported to be barriers.

*“The topography of the area is very bad. The road is hilly and stony. The river divides the villagers and the health post. It is difficult for parents to bring their children to the health post because of the river and the difficult topography.” (*WDA leader, Oromia*)*

### Supply-side barriers

The four supply side barriers identified in this study include drug stockouts, health post closure, HEWs’ lack of skill and confidence in treating sick young infants and poorly equipped health posts.

#### Drug stockouts

Interrupted supply of iCCM/CBNC commodities was a major supply-side barrier that affected the utilization of services and contributed heavily to the perception of poor-quality services at the health post. Most interviewed HEWs also acknowledged that the frequent stockouts of iCCM/CBNC commodities led to service interruption:

*“The majority of the people in the kebele have an interest in bringing their sick children to the health post, but the problem is the frequent service interruption due to inconsistent drug supply. When we get the supplies, we announce that we have the drugs, and the service is resumed. But after some time, we face drug stockouts and the service is interrupted again. As a result, the community is forced to look for other options.”* (IDI with HEW, Oromia)

District stakeholders noted that the drug stockouts happened not only due to interrupted drug supply, but also due to the limited skill of HEWs in supply chain management, as they failed to forecast and request re-supplies on time. In addition, the distribution of drugs that were close to their expiry dates contributed to the problem, according to HEWs and the stakeholders.

#### Health post closure

Health post closure was another supply-side deterrent to care seeking. Parents noted their inability to locate HEWs for emergency care during weekends or after-hours.

*"My four-year-old child was sick two weeks ago. He had diarrhea and vomiting, I gave him rice water, mixed with lemon and sugar. Unfortunately, he became unconscious...I wanted to go to the health post, but the HEWs do not work at night. So, I took him to the health center, around 7 kilometers away from my home, at night. Finally, my child recovered after taking treatment at the health center".* (IDI with mother who did not seek care, SNNPR)

Lack of safe residential houses for the HEWs forced them to live far from the health post. This in turn prohibited HEWs from providing emergency services during the weekend or at night.

*“The health post has no fence. There are no houses in the surrounding area, so we do not feel safe [to live close to the health post].”* (IDI with HEW, SNNP)

Even in places where HEWs lived within the compound of the health posts, parents complained that some of the HEWs did not open their doors to provide emergency services outside of working hours. Parents disclosed their concern that the HEWs closed the health post without adequate reason.

Apart from weekends and after-hours closures, health posts were closed when the HEWs went to the community for home visits or when they were called for meetings or training. Such interruptions were reported to have negatively affected treatment-seeking behavior.

“*It is difficult to get service in the health post, as the HEWs are sometimes not available. When we come here, they may be out in the villages...Sometimes they may go out for meetings.*” (Mother who did not seek care, FGD participant, Amhara)

Some participants noted that based on their previous experience of health post closure, they resorted to traditional medicine or to cost-incurring options.

“*We do not go to the health post as it is most of the time closed. We try home remedies. If there is no improvement, we go to private [providers], if we have the money.*” (IDI with mother who did not seek care, Oromia)

HEWs, on the other hand, noted that their engagement went beyond the health sector, which overburdened them.

*“Workload is our main challenge. When we are assigned for a political or agricultural mission out into the community, we are forced to close the health post and the mothers would not get us.”* (IDI with HEW, SNNP)

#### Lack of confidence and skill among HEWs in treating sick young infants

Most HEWs were trained on iCCM, but some HEWs had not yet received training on CBNC. Even the trained HEWs felt that they lacked the skill and confidence needed to adequately treat sick young infants.

*“It is very difficult to treat children under two months old. We need frequent refresher training and orientations.”* (IDI with HEW, Oromia)

*“HEWs can treat malnutrition, malaria, pneumonia and diarrheal diseases very well. However, they have gaps in treating sick young infants, as this is a newly acquired skill.”* (Stakeholder, SNNP)

#### Poor physical structure or lack of essential equipment at the health post

HEWs indicated that the health posts were not well-equipped, which affected their work and their ability to provide high-quality care.

“*We have no chairs [in our health post] for parents to sit on during examinations. We assess sick children while the mother is standing carrying her sick child. We also give injections and any treatment while she is standing carrying her child. We do not have benches for clients to sit on while waiting. This is challenging for us*.” (IDI with HEW, Oromia)

Community members have raised the poor physical structure of the health post and its uncleanliness as an important deterrent to care seeking.

### Suggested solutions to increase uptake of iCCM and CBNC services

The solutions suggested by the study participants are grouped into eight thematic areas.

#### Strengthening the WDA platform and enhancing awareness-raising by HEWs

Participants emphasized the importance of strengthening the WDA platform to bolster the link between communities and HEWs. They also highlighted the importance of creating awareness of iCCM and CBNC services and enhancing risk perception about childhood illnesses using HEWs and the WDA. These were effective strategies used in Degua Tembien, the best performing district in Tigray, where *kebele* cabinet members and WDA leaders worked closely with the HEWs. Their collaboration enhanced the link between the community and the health facilities, which in turn led to increased uptake of services.

*“We have regular meetings with the HEWs to discuss about different health issues...When we visit the villages, we check and report to HEWs if there are children who are sick or malnourished.”* (WDA leader, Degua Tembien, Tigray)

Mothers who were aware of the availability of iCCM and CBNC services reported that they received the information from HEWs. Some HEWs indicated that they promote the availability of services and how they can be reached by the community members when they are out for field work.

“*We have prepared and posted a banner to show the services available in the health post. Most families have school age children who can read and tell them the available services. They can also get our phone numbers from the banner and call us*.*”* (IDI with HEW, Meskan, SNNP)

#### Engaging religious leaders and using community structures

Gaining the support of religious leaders and traditional healers to address misconceptions about the causes of childhood illness was suggested to break the long-standing traditional beliefs that inhibit care-seeking. Organizing and utilizing community structures to change community perceptions and awareness was a common suggestion in all the districts. Community participants suggested several community platforms that could be used, such as churches, ‘*idir*’ (local term for community groups to help each other during funerals), ‘*ekub*’ (local term for a community savings groups) and ‘*senbete*’ (local term for gatherings of Orthodox Christian believers at church) to provide education and to mobilize the community.

*“Our society has due respect toward religious leaders … If religious fathers tell the community to take their children to health posts when they are sick, they easily listen to them rather than the health professionals.”* (Stakeholder, Amhara)

#### Using a multi-sectoral approach to create demand

Mobilizing different sectors to collaborate was suggested as an effective method to increase awareness of iCCM and CBNC programs. Participants recommended that political leaders become actively involved in championing iCCM and CBNC services to generate interest in their use.

“*To enhance iCCM and CBNC service uptake, all government structures, including youth, women, and children affairs, should work in a coordinated way.*” (Stakeholder, Amhara)

#### Establishing a feedback mechanism to address community concerns

Conducting consultative meetings with community members to identify and solve health service-related grievances was one of the strategies used by Meskan district health office.

*“We have meetings with the community every three months to identify our strengths and weaknesses, so that we keep our strengths and work to improve our weaknesses. A community complaint about service interruption at health posts is the main one. We informed HEWs to give services for 24 hours, including weekends.”* (Stakeholder, Meskan, Gurage, SNNP)

#### Ensuring the provision of iCCM and CBNC services during working hours

District managers reported several strategies to avoid service disruption when HEWs are out of the health posts. These included temporarily assigning health workers from health centers to health posts, temporarily re-assigning HEWs from places where there are two HEWs to cover for HEWs during training, and a better coordinated schedule during training or meetings, to ensure at least one of the HEWs would be available at the health post.

#### Providing iCCM/CBNC services during home visits

In Jawi, the highest-performing district in Amhara, HEWs carried iCCM and CBNC commodities during home visits, so they could carry out active case finding and treat sick children they identified.

*“Whenever I go to the field, I carry my iCCM and CBNC kit. If a parent reports that a child is sick or if I find one myself, then I treat them.”* (IDI with HEW, Jawi, Amhara)

Several participants suggested that all HEWs should adopt the practice of carrying out active case finding and treating sick children during their home visits to overcome problems related to access.

#### Building the capacity of HEWs

To increase HEWs’ capacity to provide respectful and compassionate care, stakeholders recommended that they should receive training on interpersonal communication. Stakeholder respondents also suggested training HEWs on drug supply chain management to mitigate stockouts. They also emphasized that HEWs need ongoing supportive supervision and review meetings to increase their confidence in treating newborns and children.

#### Ensuring the availability of child health commodities

Stakeholders emphasized the need to strengthen supply chain management to ensure adequate and timely provision of drugs and supplies to the health post.

## Discussion

This study found that several demand- and supply-side factors act as barriers to the utilization of iCCM and CBNC services in rural agrarian areas of Ethiopia. Deep-rooted cultural beliefs, misconceptions around the causes of childhood illnesses that led to preference to traditional healers, and a general lack of understanding of the capacity of HEWs were reported as key demand barriers to health-seeking. Drug stock-outs, erratic health post hours and closures, HEWs’ lack of confidence in their clinical skills, as well as under-resourced physical facilities were cited as key supply-side barriers. The results of this study were consistent with the results found in the previous studies on barriers to care seeking for iCCM in Oromia Region [16, 18, 19].

This study showed that misconceptions related to disease causation and risk were highly prevalent in the study areas in all four regions. Such misconception around childhood illness causation is consistent with previous studies [20–22]. The perception that childhood illnesses are inherently low risk is also consistent with previous research [23]. A survey conducted in Amhara, Tigray and SNNP by JSI/L10K showed that 42.2% of caregiver respondents cited a belief that childhood illnesses are low risk, and that the child will get better as the top rationale for not seeking care [24]. These findings indicate the need to address misconceptions related to child illness causation and risk. Participants in our study recommended use of existing community structures and influential community leaders in addition to WDA leaders and HEWs to address such long standing and highly prevalent misconceptions.

The cultural inclination towards traditional healing methods is in line with previous work and should thus be considered when designing future programs [16, 20, 24–26]. Working closely with traditional healers may help to address these deep-rooted beliefs. A study in South Africa found that traditional healers were interested in working with modern practitioners and referred patients to health facilities [27]. In a similar study that looked into local solutions to care-seeking in Kenya, educating local healers and spiritual leaders to be advocates of health facility attendance was suggested as a solution by study participants [28]. Further studies are needed to better understand how traditional healers can be recruited to encourage use of health services.

This study showed that many people did not understand the services provided at the health post. This had been shown as an important barrier in previous studies [16, 24]. This could be due to the evolving role of HEWs over time. They initially provided only preventive and promotive services. As the program matured, they began providing curative services, but the demand generation activities were insufficient. The fact that HEWs still devote most of their time to preventive activities [6, 29] may have reinforced the assumption that they cannot do much for sick children. Previous research has highlighted the importance of demand creation interventions for iCCM [19, 30].

The WDA was introduced to create a strong bridge between the health post-based HEWs and the communities. However, although around 3 million volunteers are said to be mobilized as WDA nationwide, they have not been equipped or utilized to promote child health issues effectively. WDA leaders shared most of the myths and misconceptions prevailing among the communities and lacked the understanding of the full range of services provided by HEWs. A previous study has shown that only less than half of the study population were able to identify the WDA leaders in their neighborhood [24]. Further work is needed to strengthen the WDA through building the capacity of WDA members and increasing awareness of their role in communities.

We also found key supply-side barriers that must be addressed if the iCCM and CBNC programs are to achieve their expected impact. Lower tracer drug availability at the health post has been documented elsewhere [16, 31–33]. Drug stockouts had far reaching consequences in eroding trust in HEWs and the health system. It contributed to the perceived lack of skill of HEWs to provide treatment. Stockouts reduced the likelihood that a single episode would be treated, as well as the likelihood that future episodes would be treated. Furthermore, other caregivers may be less likely to take their children to the health post because of a neighbor’s negative experience. Strengthening supply chain management to ensure adequate and timely provision of drugs and supplies, including training HEWs on the integrated pharmaceutical logistics system, will be crucial, which is in line with the suggested solution of the study participants.

Despite the official health post working hours, a common barrier to the uptake of services was health post closure. Such closures have been shown to be a major challenge in previous studies [12, 16, 29, 34]. Accountability mechanisms to ensure health posts are open and services are available during working hours needs to be established. HEWs travel for activities such as training should be staggered so that there is at least one HEW in each health post. Random spot checks by supervisors or empowering community members to report missing HEWs could be effective measures to increase accountability.

Considering the large population (average of 5000) and large geographic areas served by one health post, having HEWs spend a large amount of time on health promotion at the community level may not be very efficient. With the establishment of the WDA, a division of labor that allows HEWs to focus more on clinical activities and WDA members to focus on health promotion and community mobilization may be more practical and effective. WDA members are well placed to conduct home visits for active case finding, as well as health education. With a strong link and frequent communication between the WDA members and the HEWs, the WDA could detect cases in the community and refer them to the health post.

Even though the HEWs have received competency-based training on iCCM and CBNC, many HEWs, as well as some district and kebele health officials and community members, expressed the concern that some HEWs did not have adequate clinical skills. This was a particular concern regarding the management of sick young infants. This lack of confidence could have been aggravated by the low caseloads, which may deny HEWs the opportunity to practice their newly acquired skill in treating sick young infants. This in turn leads to a vicious cycle between the demand- and the supply-side barriers. Thus, the program would benefit from periodic on-the-job training and mentorship by the health center staff to improve HEWs’ skill and confidence. This is in line with the solution suggested by the stakeholders. This study found that not all active HEWs had been trained on iCCM and CBNC. Inclusion of the iCCM and CBNC training in the HEWs’ pre-service curriculum is important to address this gap in a sustainable manner.

Comparative analysis of the higher-performing and lower-performing districts did not show striking differences. However, HEWs in some of the higher-performing districts were able to integrate the treatment of sick children with their home visits by taking their job aids and iCCM/CBNC commodities. In Tigray, strengthened collaboration between the WDA and the HEWs was noted. These best practices should be adopted widely.

With results from the four agrarian regions of the country, this study provides a strong evidence base regarding barriers to utilization of iCCM and CBNC services in rural areas of the agrarian regions of Ethiopia. The study has assessed in-depth prevailing barriers and explored solutions from the grass root level.

This study has limitations. As is the case with all qualitative research, there is a risk of misinterpretation of results and social desirability bias. However, triangulating the results among different stakeholders helped in validating the results. Since the Health Extension Program and the WDA are evolving over time, the results of this study may not consider recent developments. In addition, these results are not representative of the other regions of Ethiopia, characterized by mainly pastoralist and urban settings.

## Conclusions

iCCM and CBNC are essential services aimed at reducing newborn and child mortality. However, misconceptions on illness causation and preference for traditional healers were found to be highly prevalent demand-side barriers. Limited awareness of the available services and perceived low quality of curative services provided by HEWs were additional important demand-side barriers. The fact that the misconceptions are shared by WDA leaders and their limited understanding of the services provided by HEWs, calls for additional work in strengthening this structure. WDA leaders need additional training to deepen their knowledge on childhood illness and causation in relation to appropriate treatment options so that they become better equipped change agents.

Geographic challenges that limited access to the health posts were the other barriers.

Inconsistent availability of services (health post closure and stockouts) along with poor confidence of HEWs to treat young infants and under-resourced physical facilities were the main supply-side barriers.

In order to increase utilization of these services, it will be necessary to address both demand- and supply-side barriers. This study highlighted the importance of demand-generation activities in any future roll-out of new community-based services. It is also important to generate adequate demand by identifying and addressing prevailing barriers that deprioritize health services. Alongside demand generation activities, supply-side barriers would need to be addressed by strengthening the health system to uphold newly introduced services to harness sustainable impact.

## Supplementary Information

**Additional file 1.**

## Data Availability

The dataset is readily available upon request with permission of PATH and UNICEF. Nesibu Agonafir can be contacted at nagonafir@path.org to access the data.

## Abbreviations

ACT: Artemisinin-based combination therapy
CBNC: Community Based Newborn Care
EPHI: Ethiopian Public Health Institute
FMoH: Federal Ministry of Health
FGD: Focus Group Discussion
HEWs: Health Extension Workers
iCCM: integrated community case management of childhood illnesses
IDI: In-depth interviews
MDG: Millennium Development Goal
MCH: Maternal child health
OHEP: Optimizing the Health Extension Program
ORS: Oral rehydration solution
SNNPR: Southern Nations, Nationalities and Peoples Region
UNICEF: United Nations Children’s Fund

## Footnotes

1 The newborn mortality rate was 29 per 1000 live births according to the EDHS, 2016.

2 PATH is an international, non-profit global health organization based in Seattle with 70+ offices around the world including Ethiopia.

3 UNICEF works within the United Nations in over 190 countries including Ethiopia to help children survive, thrive and fulfill their potential, from early childhood through adolescence.

4 Regions are administratively further divided into progressively smaller areas: zones, *woredas* (districts) and *kebeles* (sub-districts).

5 As most residents in the Gurage zone speak Amharic, the data collectors used the Amharic data collection tools and translated it to Gruagina during the interview as needed.
