# Supplementary material for: Barriers to the uptake of community-based curative child health services in Ethiopia
Source: BMC Public Health. 2021 Aug 14;21:1551. doi: 10.1186/s12889-021-11558-2 (PMC8364059; doi:10.1186/s12889-021-11558-2)
Supplement: Supplementary file 1 — Additional file 1. [file 12889_2021_11558_MOESM1_ESM.docx]

# Table

## Table 1: Study area

| Region | Zone | Number and name of sampled districts |
| --- | --- | --- |
| Amhara | Awi | 4 (Banja, Fagta, Ankesha, Jawi*) |
| Oromia | Guji | 2 (Wadera, Saba-Boru) |
|  | West Hararge | 2 (Boke, Doba*) |
| SNNP^[[1]](#footnote-1)^ | Gurage | 4 (Geta*, Meskan, Gummer, Abeshige) |
| Tigray | South Eastern | 3 (Enderta, Degua Tembien*, Hintalo Wajirat) |
| Total | 5 | 15 |

*Higher-performing districts

## Table 2: Data collection methods and participants per districts

| Participant Type | | Number of In-depth Interview per district | Number of focus group discussion per district | Average number of participants | Total number of participants | Age range |
| --- | --- | --- | --- | --- | --- | --- |
| Mothers | Who did not seek care for their sick children from health post | 2 | 1 | 8.4 | 126 | 18-48 |
|  | Who sought care for their sick children from health post (exit interview whenever possible) | 1 |  | 1 | 15 | 20-65 |
| Fathers | |  | 1 | 8 | 120 | 20-62 |
| *Kebele* (sub-district) Cabinet Members | |  | 1 | 6.3 | 95 | 22-55 |
| Health Extension Workers | | 1 |  | 1 | 15 | 21-36 |
| Women Development Army Leaders | |  | 1 | 7.8 | 117 | 18-69 |
| Community leaders | |  | 1 | 6.6 | 99 | 25-90 |
| District level stakeholders | |  | 1 | 5.1 | 77 | 22-54 |
| Total | | 4 | 6 | 44.4 | 664 | 18-90 |

1. Southern Nations, Nationalities and Peoples Region [↑](#footnote-ref-1)
